# The Work of Mechanical Degradation in Elongating Polymer Melts

**DOI:** 10.1021/acs.macromol.4c02063

**Published:** 2025-02-14

**Authors:** Nattavipa Chongvimansin, Thomas C. O’Connor

**Affiliations:** Department of Materials Science and Engineering, Carnegie Mellon University, Pittsburgh, Pennsylvania 15217, United States

## Abstract

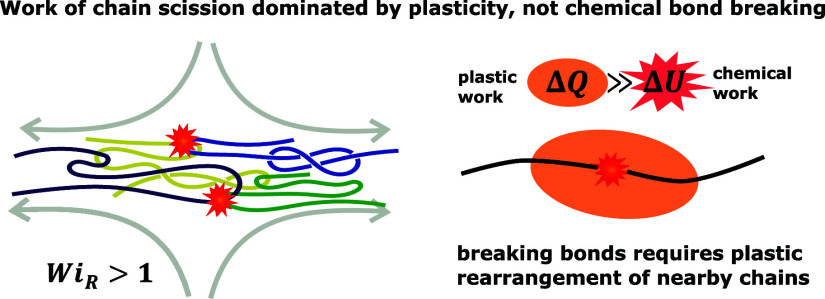

Molecular dynamics simulations are used to study the mechanical degradation of well-entangled polymer melts during uniaxial extensional flow. Simulations measure the transient rise in extensional stresses and relate them to the molecular alignment and scission of chain backbones. Intermolecular entanglements couple chain scission in space and time, making degradation sensitive to deformation history and strain rate in ways not displayed by dilute polymer solutions. The rate of chain scission is nonmonotonic and peaks at strains corresponding to the maximum extensibility of entanglement segments but prior to the full extension of chain backbones. We measure a specific work per scission event w* and decompose it into separate contributions associated with chain alignment, chemical bond breaking, and scission-induced plasticity. We find chain scission in melts requires activating plastic dissipation that is multiple orders of magnitude larger than the chemical work required to break a covalent backbone bond. Our findings underscore the critical need to consider bulk polymer mechanics and rheology in designing efficient mechanical degradation and mechanochemical processes.

## Introduction

The outsized need to manage, degrade, and repurpose plastic waste has focused attention on methods for breaking down long polymers into more easily processed forms.^1−3^ While many are exploring novel chemical and catalytic processes for degradation, the simplest and most accessible way to break down chains is through mechanical degradation, where chains are elongated by flow and break under tension. However, most polymer processing research has focused on avoiding mechanical degradation in molten polymers. As such, a full physical model for mechanical degradation in concentrated polymers remains unknown, but these models are needed to make mechanical reprocessing viable at industrial scales. Concentrated polymers exhibit complex viscoelasticity, and degradation of chains through scission can dramatically alter their fluid properties.^4,5^ Without a mechanistic understanding of these behaviors, reprocessing flows cannot be reliably designed and controlled.

Most systematic studies of flow-induced chain scission have focused on dilute solutions of chains subjected to planar or uniaxial extensional flows. Experimental and numerical investigations^6−10^ have shown that polymer chain scission can be induced when flows elongate dilute chain conformations.^7,8,11,12^ These studies have produced reasonably accurate predictions for degradation kinetics and products for isolated chains where scission events are uncorrelated. They have shown that the scission of dilute chains can be modeled as a thermo-mechanically activated process where scission is biased by tension in the polymer backbone.^6,9,13,14^ This causes chains to preferentially break near their center where tension is the highest, which has been observed in both experiments and simulations.^7−9,15−18^ They also predict a flow-rate-dependent exponential decay in the fraction of intact chains ϕ_N_ at a fixed strain rate.

While dilute solutions exhibit simple degradation kinetics, dilute polymers are not cost-effective for practical recycling due to their low yield and low mechanical efficiency. This motivates our study on flow-driven mechanical degradation in entangled melts. Polymer melts display much more complex stress relaxation and chain dynamics during flow due to chain entanglement,^19,20^ and a general understanding of how entanglements mediate chain scission remains unknown.^21−23^ Unlike solutions, chain alignment in melts is mediated by the entanglement network, causing chains to undergo a relatively slow unraveling process before they can fully elongate with the flow.^24^ However, the hidden topological structure is almost inaccessible to experiments, especially during rapid nonlinear flows. As we will show, this process has a profound impact on mechanical degradation.

Researchers have long been interested in learning how to avoid chain scission during polymer melt processing. Prior studies have comprehensively investigated how extensional deformation can drive chain scission in melts undergoing filament stretching.^25−27^ A systematic analysis of the conditions under which chain scission occurs in filament extension is well summarized in a review article by Malkin and Petrie.^28^ However, they observe that chain scission is difficult to maintain during filament stretching due to surface-mediated failure of the thin filament geometry. To avoid this, others have studied chain scission during converging flows through microfluidic cells which have no free surfaces, but these studies were limited to semidilute polymer solutions.^10^ Molecular Dynamics (MD) simulations provide an alternative means to study chain scission during strongly nonlinear flows. Recent advances in nonequilibrium MD permit simulating entangled melts in strongly nonlinear flow conditions. Applications of this approach have successfully reproduced the rate-dependent nonlinear viscoelasticity of molten polymers with varying chain architecture and chemical interactions.^29−33^ Notably, MD simulations can resolve bulk extensional flow behavior to arbitrarily large strains and strain rates without the presence of free surfaces or surface instabilities. This enables MD simulations to probe the behavior of polymers undergoing strong extensional flow conditions within a closed continuous flow geometry.

In this study, we apply nonequilibrium MD simulations to investigate flow-induced chain scission of entangled polymer melts undergoing bulk uniaxial extensional flow. We measure the evolution of the extensional stress in relation to molecular alignment and chain scission. In contrast to dilute solutions, which exhibit a constant chain scission rate, our melts demonstrate a history-dependent scission rate that deviates from a simple thermally activated process.^14^ By quantifying the specific work per scission event, we show that plastic dissipation dominates over the chemical energy required to break bonds. Our findings underscore the critical role of chain entanglements and viscoelastic dissipation in mechanical degradation processes.

## Models and Methods

We model polymers with a Kremer-Grest bead–spring model^34^ modified to permit the breaking of backbone bonds. All monomers have mass *m* = 1, and interact through a Lennard-Jones (LJ) potential with energy scale ϵ, length scale σ, and potential cutoff σ^2^1/6^^. All quantities in this paper are expressed in reduced LJ units with base units of energy ϵ, length σ, and time . We connect monomers into *M* = 400 chains of length *N* = 400 beads with *N* – 1 backbone bonds with a mean bond length *b* = 0.96. The backbone stiffness is set by applying a bond angle potential beween adjacent bonds *U*_a_ = *k*_θ_(1 – cos θ), where θ is the angle by which the bonds deviate from parallel alignment. To ensure that angular interactions are removed upon bond breakage, we implement a modification to the LAMMPS source code, permitting the force field to dynamically exclude angular interactions after bonds dissociate. These LAMMPS modifications are available through the O’Connor Lab’s GitHub page at https://github.com/OConnor-Lab.

Bonds are modeled either with an unbreakable FENE potential or a breakable quartic potential defined as ϕ(*r*) = *K*(*r* – *R*_c_)^2^(*r* – *R*_c_ – *B*_1_)(*r* – *R*_c_ – *B*_2_) + *U*_0_. Following prior studies, Ge et al., we set *R*_c_ = 1.5 and *B*_2_ = 0 such that the cohesive energy of a bond .^35^ This study considers two systems with *K* = 1300 and 1500 which give cohesive energies *U*/*k*_B_*T* ≈ 48 and 41. Since our model energies are measured relative to the van der Waals bond energy ϵ, *U* is expressed as the ratio of the covalent bond energy to the van der Waals bond energy, which is typically ∼10–100 for common polymer chemistries like polyolefins.^36^ In choosing our bond potential parameters, we verify that *U*/*k_B_T* is large enough that covalent bonds are stable in equilibrium and do not spontaneously break due to thermal fluctuations at *T* = 1.

We equilibrate our melts at a monomer number density ρ = 0.85 and *T* = 1 by applying a Nosé–Hoover thermostat. The thermostat damping time varied, but was chosen to be larger than the time scale for particle collisions as measured by the decay of the monomer velocity autocorrelation function. Prior studies have shown that this density and temperature produces an entangled polymer melt with a chemical entanglement length *N*_e_ ≈ 28 and entanglement relaxation time τ_e_ = 1.98 × 10^3^τ_LJ_.^30,37,38^ For our chain length *N* = 400 this produces a melt with a degree of entanglement *Z* = 14 which displays nonlinear rheology similar to atactic polystyrene melts.^29,38^

Uniaxial extensional flows are simulated by applying Generalized Kraynik Reinelt (GRK) boundary conditions and integrating the g-SLLOD equations of motion in LAMMPS.^39^ Systems are stretched along the *z*-axis at constant Hencky strain rate ϵ̇_H_ with Rouse Weissenberg numbers *Wi*_R_ = ϵ̇_H_τ_R_ ranging from 40 to 404. The Rouse relaxation time of the entangled polymers τ_R_ is the characteristic time it takes for polymer chains to relax to their equilibrium configurations.^40^ The systems are run until they reach a final Hencky strain ϵ_H_ = 4.0, which is comparable to the largest strains achieved in filament stretching experiments.^41^ By comparing systems with both unbreakable and breakable chains in similar flows, we can determine the impact of mechanical degradation on the nonlinear rheology.

During start-up flow, we measure the evolution of both the macroscopic extensional stress σ_E_(*t*) = σ_*zz*_ – σ_rr_ and the microscopic history of all chain scission events for every 0.1 strain output. σ_*zz*_ denotes stress acting in the axial (*z*-axis) and the radial direction of the cylindrical coordinate system is defined by .

Given that chains can break, resulting in polydisperse melts, careful consideration was given to the statistics of the remaining unbroken chains (*N* = 400). Thus, chain extension is measured by computing the average extension ratio *h*(*N*_e_) = *R*(*N*_e_)/(*N*_e_)⟨*b*⟩ for *unbroken* chains. *R*(*N*_e_) is the average RMS end-to-end distance for a segment of *N*_e_ beads and ⟨*b*⟩ is the average bond length for each chain during flow. *N*_e_ is the distance over which polymer chains become physically intertwined, which is referred to as an entanglement length. Lastly, the number of chain scissions (*N*_b_) is determined by keeping track of the number of bonds at each time interval.

## Results & Discussion

**Figure 1:**
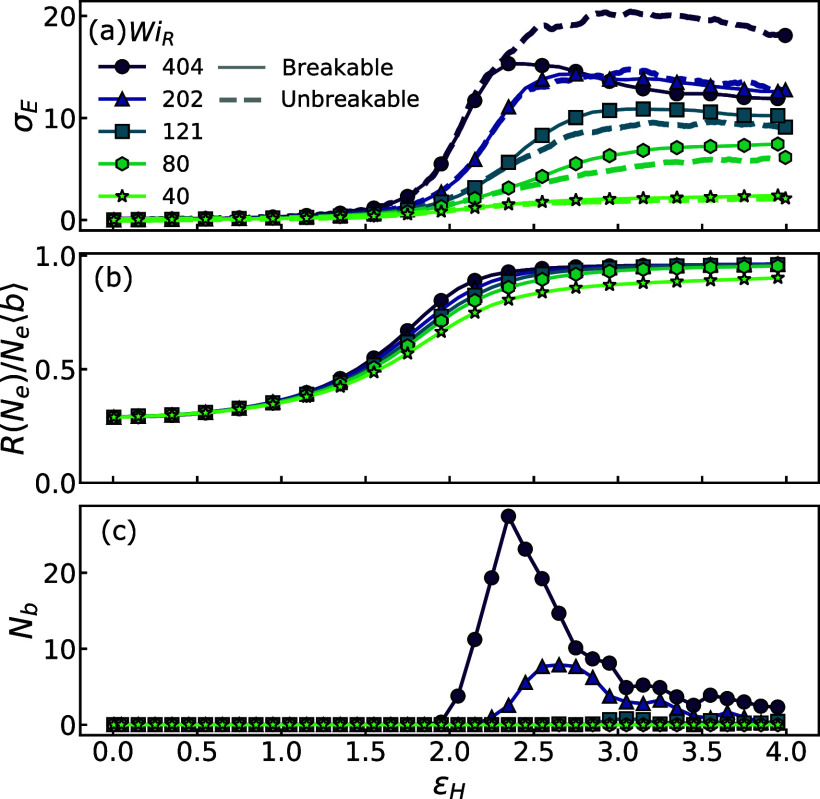
(a) The macroscopic extensional stress σ_E_. The solid line depicts σ_E_ from breakable polymer systems, while the dashed line corresponds to σ_E_ from unbreakable systems. (b) The average entanglement extension *R*(*N*_e_)/*N*_e_⟨*b*⟩. (c) The frequency of bond scission *N*_b_, as a function of strain for *Wi*_R_ = 40–404 and *U*/*k*_B_*T* ≈ 48.

Figure 1a plots σ_E_ versus the Hencky strain for melts with both breakable (solid lines) and unbreakable (dashed lines) backbones, (b) plots the extension ratio at the entanglement length scale *h*(*N*_e_), and (c) plots the corresponding *N*_b_ within each interval of strain. Represented in this way, we can clearly see how microscopic chain scission events correlate with the time evolution of the extensional stress and chain tension.

In the initial stage of flow (ϵ_H_ < 2.0), the entanglement network begins to align. During this stage, chain extension remains minimal, and no chain scission is observed. Upon deformation, the stress function σ_E_(*t*) exhibits a sharp increase around the strain threshold of ϵ_H_ ≃ 1.5–2.0. This corresponds with the nearly complete alignment of the entanglement network and the stretching of entanglement segments to a scale of approximately *N*_e_. This produces significant local tensions and triggers a rapid increase in chain scission.

Due to the high rate *Wi*_R_ ≫ 1, chains deform almost affinely with the flow, and *h* grows similarly with ϵ_H_ at all *Wi*_R_ with a slight enhancement in stress for higher *Wi*_R_. As σ_E_ increases, curves develop a maximum stress during the initial stress rise. This maximum cannot be solely attributed to the viscoelastic stress associated with aligning chains since the extension ratio of chain backbones *h*(*N*_e_) displays a monotonic increase, as shown in Figure 1b.

Instead, the stress peak arises from the work associated with chain scission. This is evident in Figure 1c which plots the number of bond scission events per unit strain (*N*_b_) and shows a peak in bond breaking corresponding with the peak in σ_E_. At lower strain rates (*Wi*_R_), the difference in stress between the breakable and unbreakable systems diminishes, with correspondingly fewer scission events. These results indicate that chain scission in polymer melts coincides with a stress peak and requires excess work beyond aligning polymer backbones. This suggests bond breaking involves the plastic deformation of surrounding molecules.

Previous studies of mechanical degradation in dilute polymer solutions have measured scission kinetics via the fraction of unbroken chains ϕ_N_(*t*) = *M*(*t*)/*M*_0_ where *M*(*t*) is the number of surviving chains with the original length *N* = 400 at time *t* and *M*_0_ is the original number of chains.^6,9,13,14^ In dilute systems, ϕ_N_(*t*) develops a constant exponential rate of decay in steady-state flow, with a rate that depends upon the strain rate. This corresponds to chains extending and breaking independently at a constant rate in time and can be modeled as a stress-biased thermally activated process. From Figure 1c, it is clear that the melts do not develop a steady rate of bond scission, even though Figure 1b shows a steady-state tension in chain backbones. Instead, the melts show a maximum rate of scission during the initial rise in *h*(*N*_e_), which then slows down as chains unravel and extend at larger scales. We observe this trend in all systems where scission occurs. These trends indicate that chains in melts are more prone to breaking during the initial stages of unraveling from the entanglement network when their backbones are locally taut but still highly entangled with other chains. This burst of scissions degrades the entanglement network, trapping some chains in folded conformations that resist unraveling. As the network deteriorates, the transmission of macroscopic strain to individual chains becomes less efficient. On the other hand, chains unravel and become fully aligned at low *Wi*_R_. Once their entanglement points are removed, local stresses along the chain backbones decrease, leading to a reduced rate of degradation. This aligns with Desai and Larson’s findings on entanglement unraveling^42^ and Ianniruberto et al.’s work on friction reduction.^43^

**Figure 2:**
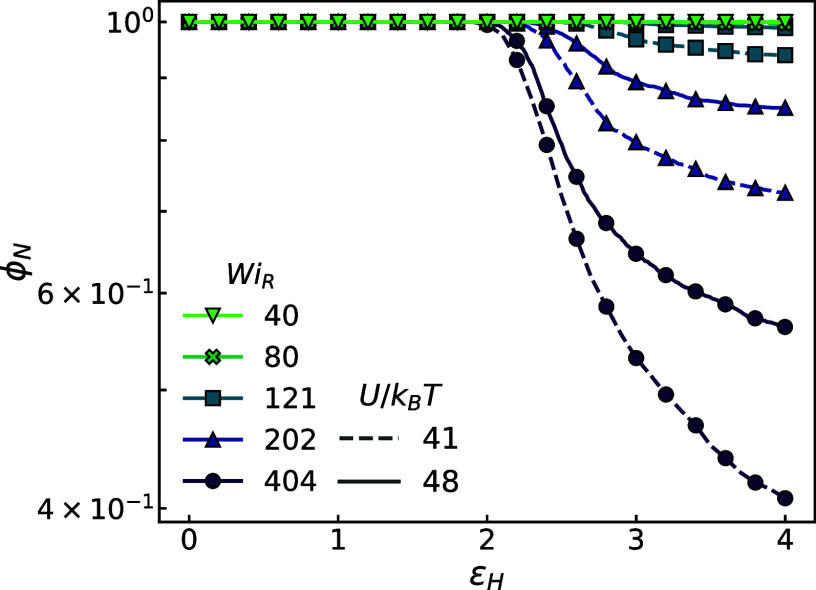
Scission kinetics (ϕ_N_) across strain ϵ_H_ for simulated systems in extensional flow, ranging from *Wi*_R_ = 40 to 404, with *U*/*k*_B_*T* ≈ 41 and 48 as specified in the legend.

Figure 2 shows ϕ_N_(*t*) curves for melts with two different backbone strengths *K* elongated at five different strain rates. Varying backbone strength by ∼15% can significantly alter the extent of degradation at a given strain rate but does not alter the overall trend in the kinetics. As expected, ϕ_N_(*t*) curves display an induction period during initial alignment until ϵ_H_ ∼ 2 and then rapidly degrade, but with a rate of degradation that decreases continuously as flows persist. These complex kinetics cannot be captured by a single thermally activated process as is done for solutions. As shown in Figure 2, ϕ_N_ on a log scale does not follow a simple exponential slope but slows down over time. Rather, more complex models are required which can account for the mechanical conditions and intermolecular correlations that entangled chains experience as they unravel during flow. A notable example in the literature is the recent work of Moghadam et al. on chain unraveling in elongating polymer melts.^24^

**Figure 3:**
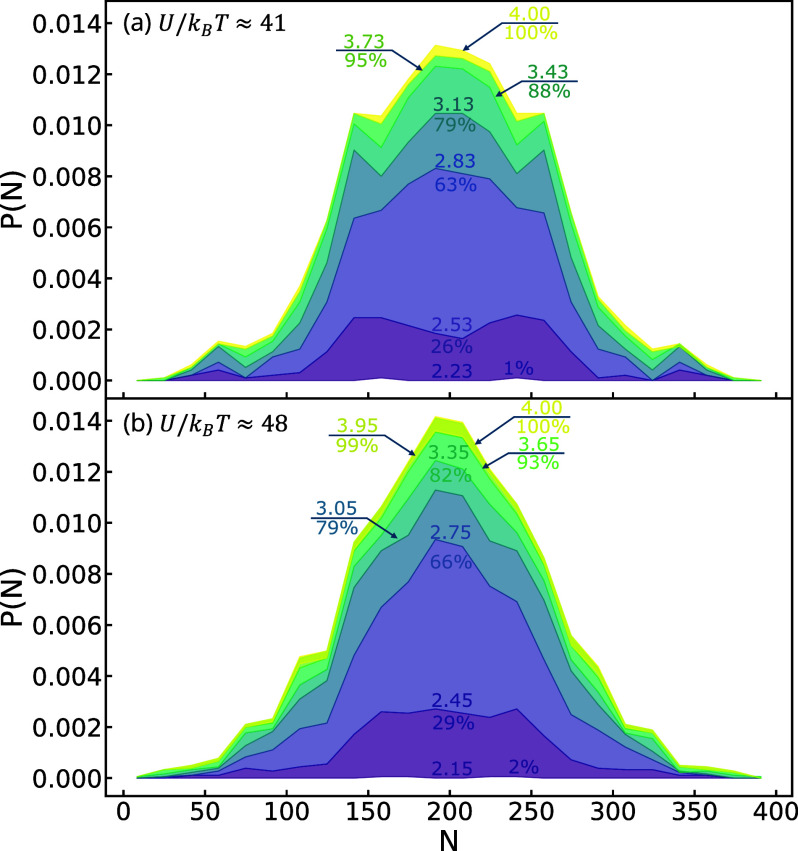
Smoothed cumulative distributions of degradation products measured in MD simulations for *Wi*_R_ = 202 and *U*/*k*_B_*T* ≈ 41 (a) and 48 (b). The numbers labeling distributions indicate the strain values and percentage of total breaking events accumulated at that strain. The plots are normalized relative to the final state such that only the final distribution is normalized.

Figure 3 shows the evolving molecular weight distribution of broken chains for both the *U*/*k*_B_*T* ≈ 41 and 48 systems at *Wi*_R_ = 202. The cumulative distributions are shown at strain increments of 0.3, up to a final strain of 4.0. The curves at earlier strains are scaled based on the number of scission events relative to the total number observed. They capture how the frequency of chain scission evolves over time. When a chain breaks at position *n*, it produces two segments: one of length *n* and the other of length *N* – *n*. This segmentation leads to the observed bimodal distribution, as the lengths of the two segments form two distinct populations. The product distributions are centered around *N*/2, similar to dilute solutions, but both the accumulation of scission products and the shape of the distributions are markedly different for melts. For the cases shown in Figure 3a,b, the majority of scission events (∼70%) occur in the strain interval ε_H_ = 2.0–3.0.

Comparing distributions in this interval reveals that these scission events are accumulating *uniformly* in a region near chain centers, producing flat-topped distributions of products. Our results suggest that the oriented entanglement network produces a region of uniform backbone tension centered around chain midpoints, leading to a large number of breaking events uniformly distributed around *N*/2. We suspect that the breadth of this region is sensitive to the entanglement properties of molten chains.

The final shape of the product distributions depends on the strength of the chain backbones and the total strain accumulated after the initial burst of degradation. Subsequent strain drives chains to unravel from the entanglement network and elongate, leading to a slower overall rate of chain scission events that distribute in a more Gaussian-like way relative to the chain center.

Comparing the two systems in Figure 3a,b shows that the weaker chains in (a) display a much flatter final distribution at a terminal strain of 4.0. These differences can be attributed to the fact that the majority of scission events in the weaker melt occur during the initial alignment of the entanglement network, where the distribution of events is more uniform across the backbone. In contrast, the stronger chains in (b) accumulate a significantly larger fraction of their total chain scission at large strains, after chains have unraveled and elongated in the flow. These elongated conformations display higher tension at their chain centers, causing the initially flat-topped distribution to develop a prominent peak at *N*/2 at large strains.

For sufficiently high *Wi*_R_ the flow is sufficiently strong to drive secondary and tertiary breaking of already broken chain segments into even smaller segments. These higher-order scission events are centered around the midpoints of their associated chains and thus lead to secondary features in *P*(*N*) near *N*/4 = 100 and *N*/8 = 50, as seen for the weaker backbone system in Figure 3a. If *Wi*_R_ is reduced or the backbone integrity is increased, these features are not observed and *P*(*N*) displays a single peak centered around *N*/2, as shown in Figure 3b.

To understand the mechanistic contributions to the work of degradation, we decompose the extensional stress into three terms σ_E_ = σ_S_ + σ_U_ + σ_Q_. The first term σ_S_ is the irreversible entropic work associated with elongating chain conformations. The second σ_U_ is the reversible chemical work of straining and breaking backbone bonds. The third term σ_Q_ is the irreversible plastic work triggered by breaking backbone bonds. Note, σ_S_ is the dissipative polymeric stress that we typically associate with melt viscoelasticity, while σ_Q_ is the work of plastic dissipation specifically triggered by bond scission during flow. It is common to assume σ_S_ and σ_U_ dominate chain scission and mechanochemistry and to ignore σ_Q_. As we will show, this cannot be done in concentrated melts where σ_Q_ is substantially larger than σ_U_.

To decompose the stress, we apply the first law of thermodynamics, , to separate the macroscopic work *dW* into reversible and irreversible terms. In our systems, this work is performed by the application of the uniaxial strain, resulting in a stress:
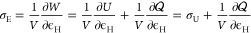
1

We further decompose the total heat dissipation into two terms: , where *s*_p_ is the polymer configurational entropy and d*q* is the excess dissipation due to bond scission that is not captured by *s*_p_. Here, lowercase symbols indicate intensive densities — e.g., *s*_p_ = *S*_p_/*V*.

This defines two dissipative stress components σ_S_ = *T*∂*s*_p_/∂ϵ_H_ and σ_Q_ = ∂*q*/∂ϵ_H_, respecitively. Both the polymer entropy *s*_p_ and internal energy *u* are functions of the microscopic coordinates of the chains and can be measured during MD simulations. Thus, our strategy will be to measure σ_S_ and σ_U_ during mechanical degradation flows, which can then be subtracted from the total extensional stress σ_E_ to estimate the unknown σ_Q_.

Following our prior studies,^29−32^ σ_S_ in flowing melts is well approximated by the Langevin entropy of entanglement segments σ_S_ = ρ*k*_B_*T*/*C*_*∞*_⟨*h*(*N*_e_) *L*^–1^(*h*(*N*_e_)) *P*_2_(*N*_*e*_)⟩, where *L*^–1^ is the inverse Langevin function and *P*_2_(*N*_e_) is the nematic orientational order parameter of entanglement segments.^44^ O’Connor et al. have shown that this expression can accurately capture the steady-state extensional stresses of melts in oriented states over a wide range of *Wi*_R_, but it tends to overpredict stresses as chains near full extension (*h* → 1) since the inverse Langevin function diverges to infinite stress in this limit.^29^ Thus, here we will treat this expression as an upper bound on the stress associated with chain alignment during startup flow.

The stress associated with the chemical work of scission σ_U_ arises from strain-induced changes in the internal energy of covalent bonds and bond angles. This stress is computed by summing the total internal energy associated with chemical bonds and angles as a function of strain *U*(ϵ) during the MD simulation and numerically taking the derivative with respect to strain via a first-order finite difference. We note that there are other sources of internal energy associated with van der Waals interactions and kinetic energy, but none change significantly since our systems are deformed at constant volume and temperature. As systems elongate, the bond energy increases as bonds stretch, but bond angle energies decrease because the angle potential prefers chains to be straight. Thus, these two energies trend in opposite directions as chains align.

Given σ_S_ and σ_U_, we can set a bound on the irreversible plastic work of scission σ_Q_ by subtracting the preceding stress terms from the total stress: σ_Q_ = σ_E_ – σ_S_ – σ_U_. Conservatively, σ_S_ sets an upper bound on polymer entropic stresses,^29^ so we consider σ_Q_ to be a *lower bound* on the plastic dissipation triggered by scission events. Even as a lower bound, we find that σ_Q_ is substantially larger than the chemical work.

**Figure 4:**
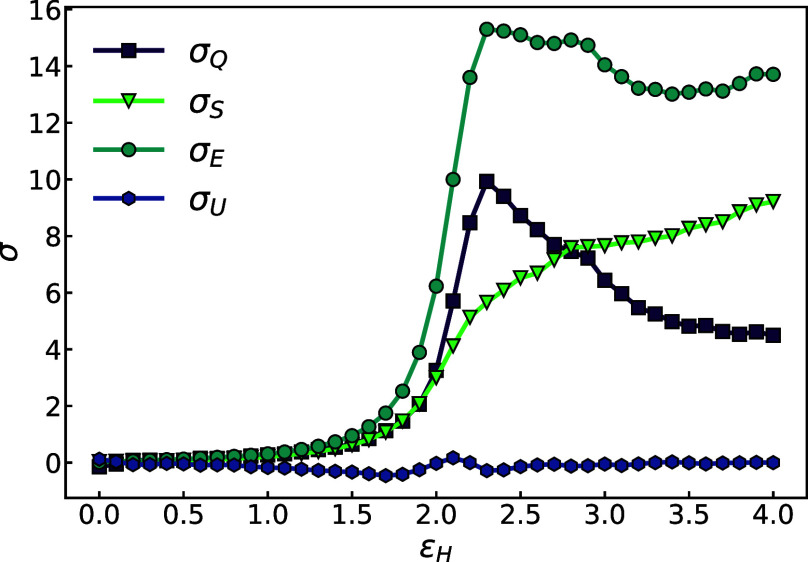
Evolution of the four stress terms over strain ϵ_H_ for *U*/*k*_B_*T* ≈ 48 at *Wi*_R_ = 404.

Figure 4 plots the evolution of each stress term versus ε_H_ for a representative melt with *U*/*k*_B_*T* ≈ 48 and *Wi*_R_ = 404. The configurational stress σ_*S*_ increases rapidly between ϵ_H_ = 1.5–2.0 in correspondence with the elongating chain conformations in Figure 1b, before slowing gradually toward a plateau at higher strains. We note true steady-states are unlikely to be achieved since degradation is constantly changing the molecular weight distribution of the melt. At the terminal strain, σ_S_ is the largest contribution to σ_E_, contributing more than 60% of the overall stress. In general, the majority of the work goes into establishing and maintaining chain alignment.

The scission stresses, σ_U_ and σ_Q_, are nonmonotonic and peak near a strain ε ≈ 2 where the rate of bond scission is maximum (Figure 1c). The chemical and plastic stresses have a striking difference in magnitude, with σ_Q_ peaking at a value 1 order of magnitude larger than σ_U_. Roughly speaking, this means most of the work associated with breaking a bond in the aligning melt is due to the plasticity triggered by displacing the covalent bond and not by the breaking of the chemical bond itself. Indeed, the chemical work of bond breaking is a negligible contribution to the total work in this system, and this trend holds for other rates and backbone ceiling temperatures. This observation runs counter to the current focus researchers place on chemical energy barriers when analyzing mechanochemical activation in polymers.^45,46^ While focusing on chemical energy changes may be appropriate when considering chains in isolation (or possibly in dilute solutions), our results predict that plastic dissipation should dominate mechanochemical scission in a dense melt environment. The relative contributions of these stress terms are maintained for varying flow rates and chain backbone strength.

To compare the overall efficiency of the degradation process across different systems and flow conditions, we compute the ratio w* = *w*_tot_/*n*_b_*U*. Here, *w*_tot_ represents the total work of the flow obtained by integrating the stress–strain curve, and *n*_b_ = *N*_b_/*V* is the total number density of scission events that occurred during the entire flow. The ratio w* quantifies the excess work required to break a bond beyond its covalent bond energy *U* and normalizes for the varying amount of degradation produced by different flows (Figure 2).

**Figure 5:**
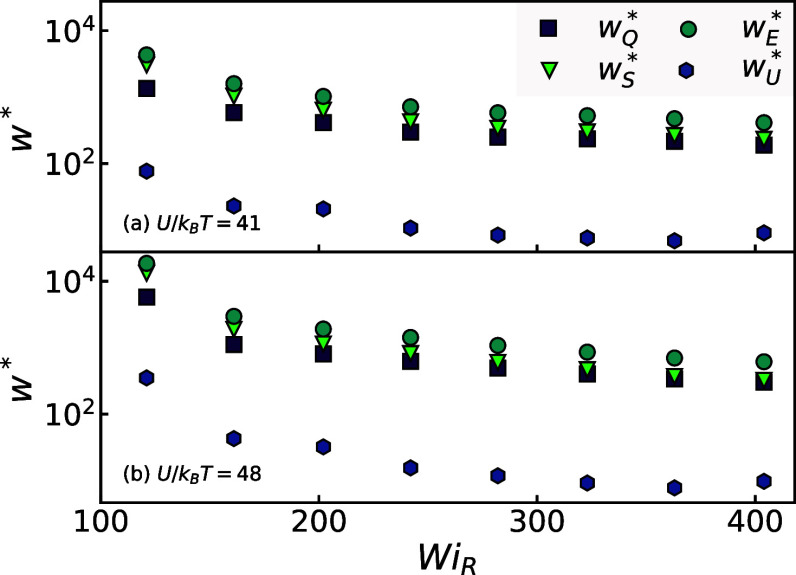
Energy cost for chain scission relative to constant chemical work (w*) across all *Wi*_R_ for the macroscopic work (*w*_E_^*^), the plastic work of scission (*w*_Q_^*^), the work of maintaining chain alignment (*w*_S_^*^), and the chemical work (*w*_U_^*^). Two backbone strengths are displayed for *U*/*k*_B_*T* ≈ 41 and 48. Data points corresponding to flow rates without chain scission are omitted.

Figure 5 plots w* versus *Wi*_R_ for both melt systems. Despite a ∼10% difference in the cohesive energy of their backbone bonds, this results in a dramatic change in degradation efficiency, lowering w* by a factor of 5 from w* ≈ 2 × 10^4^ to ∼4 × 10^3^ at the lowest strain rate *Wi*_R_ = 121. Notably, w* ∼ 10^4^ indicates that the extensional flow process requires ten thousand times the cohesive energy of one backbone bond to yield one scission event. The efficiency improves with increasing strain rate, and the curves w*(ϵ̇) for the two different melts converge, differing only by a factor ∼2 at *Wi*_R_ ∼ 400. We attribute this convergence to the molecular environments of the two melts becoming very similar at the highest strain rates, making efficiency differences more dependent on small variations in backbone strength rather than local plasticity or chain alignment. Confirming this will require further investigations.

To understand this rate dependence, we decompose the work  as we did for the stress and plot each component in Figure 5 versus rate. Similar to the stresses in Figure 4, the majority of  is due to the work of maintaining chain alignment *w*_S_^*^ followed by the plastic work of scission *w*_Q_^*^ and the least is due to chemical work *w*_U_^*^. This order is maintained for all strain rates and for both backbone strengths we show here. In all flows, *w*_U_^*^ contributes ∼1% or less to the total work required, and *w*_Q_^*^ is larger than *w*_U_^*^ by an order of magnitude or more.

The work decomposition reveals several informative trends. Despite being the smallest contribution, the chemical work is still substantial, much larger than unity, with *w*_U_^*^ ∼ 10^2^ at lower rates decreasing to ∼10 at high rates. This indicates that for every bond broken, ∼100*U* of work goes into stretching (but not breaking) other bonds in the melt. This is intuitive since the entanglement network must load whole backbones in tension before triggering scission events. The rapid drop in *w*_U_^*^ with rate indicates bonds are breaking before tension can be distributed along chain backbones into other bonds. This enhanced plastic localization with increasing strain rate agrees well with understood trends for amorphous materials.^47,48^

Comparing *w*_Q_^*^ and *w*_S_^*^, we see that *w*_S_^*^ > *w*_Q_^*^ at *Wi*_R_ = 112, but the two converge and become approximately equal at *Wi*_R_ ∼ 400. We observe this for both model chemistries. In other words, the dissipation required to break each bond becomes equal to the dissipation required to maintain the overall chain alignment for each bond-breaking event. Combined with the fact that *w*_U_^*^ is much smaller, this means that *w*_Q_^*^ and *w*_S_^*^ each contribute half of the total excess work at a high rate. The convergence of dissipation from local scission events with that required to maintain bulk chain alignment is notable, though the underlying reason remains unclear. Nevertheless, it is evident that plastic processes dominate the work of degradation and can be most effectively manipulated by varying flow rates.

The large magnitude of the dissipative work triggered by bond scission, but absent without it, implies that scission activates plastic rearrangment in a substantial region around the breaking bond. This makes sense since scission requires pulling two atoms apart and other nearby atoms must displace to accommodate this separation. For a chain in a molecular solvent, the small solvent molecules can easily accommodate a breaking chain through a local reconfiguration, but in a polymer melt the chain connectivity should significantly restrict local motions and require more cooperative plastic rearrangments and thus more dissipation. Another important contribution to the plasticity is the entanglement between chain segments. Recall that the majority of scission events occur at strains ∼2 where the local entanglement network has aligned, but chains remain highly entangled and have not yet unraveled at the end-to-end scale. As a result, the mechanical state of any given chain backbone is strongly correlated with the mechanical states of the chains it is entangled with. Thus, breaking one chain backbone can produce a cascade of intermolecular plasticity as neighboring entangled segments redistribute the tension released by the breaking segment.

**Figure 6:**
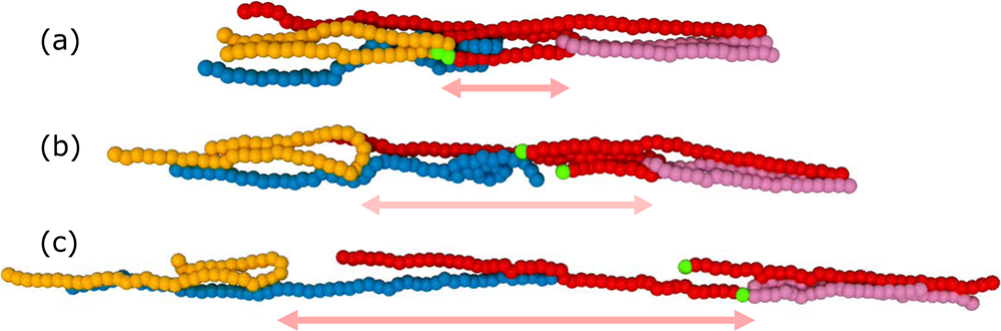
Configurations of a cluster of entangled chains undergoing a scission event in a melt with *U/k_B_T* = 48 and *Wi_R_* = 404. (a) The cluster of chains at ϵ_H_ = 2.3 just before the red chain undergoes scission at the bond marked by neon green beads. (b) and (c) show the cluster at Δϵ_H_ = 0.1 and 0.2 postscission. Double arrows indicate the displacement of the yellow and pink chains following the breakage of the bond between the green beads. For clarity, only segments of approximately 50 beads are depicted for each chain.

Figure 6 illustrates a cooperative plastic event triggered by a chain breaking in the *U/k_B_T* = 48 system at **Wi*_R_* = 404 and at a strain of ϵ_H_ ∼ 2.3. This event is selected as a "typical" event since it occurs at the most common strain for scission and on chain that has an extension ratio equal to the average extension ratio at that strain. Panels (a–c) show the atomic configuration of chain (red) and the segments it is entangled with (other colors) immediately prior to, during, and immediately after a bond has broken, respectively. The three configurations are separated by Δ*t* = 100τ_e_ corresponding to a macroscopic strain of Δϵ_H_ = 0.1. The red chain is deeply entangled with the yellow and pink chain, forming a red loop joining the three that is under high tension. The scission event indicated by bright green monomers in Figure 6b,c occurs at the sharpest bend in the loop. Immediately after the loop breaks, a massive recoil is observed in (b) and (c), with the yellow and pink loops separating much larger distances than required by the macroscopic strain rate. These large nonaffine motions of several chains certainly generate substantial dissipation. It is also likely that the recoil of these neighboring chains leads to further nonaffine motions of other unvisualized chains with which they are entangled. We aim to more thoroughly characterize these scission-induced plastic cascades in our future work.

The backfolded conformations in which chains undergo scission are diverse and difficult to relate to an average stress or chain tension. Prior studies on dilute chains have shown that the specific conformational path a chain takes to elongate can change the peak tension experienced by the chain backbone.^49−51^ The presence of entanglements further complicates this history dependence by coupling the conformational histories of different chains through entanglements. Modeling the details of this unraveling process and the segmental tensions it produces is not straightforward, but recent theoretical studies by Moghadam, Dalal, and Larson have produced promising reduced-order mechanical models for the unraveling of entangled chains during extensional flows.^24^ We suspect incorporating backbone scission into these models may be a viable route toward predicting mechanical degradation in elongated melts.

## Conclusions

We’ve applied nonequilibrium molecular dynamics simulations to model the process of mechanical degradation in elongated polymer melts. Our simulations predicted an inevitable energy loss due to plasticity induced by chain scission. While uniaxial flow is not the most practical flow for mechanical degradation processes, it is the most effective flow geometry for loading chains in tension. Any other flow geometry will necessarily introduce vorticity which will weaken the overall extension of polymer chains and result in a lower efficiency. As such, we think uniaxial flow sets a useful upper bound on the realizable efficiency of mechanical degradation processes.

We observe that intermolecular entanglements alter the kinetics of chain scission in melts, making them sensitive to deformation history and strain rate in ways not displayed by dilute solutions. The scission rate is nonmonotonic and peaks at a strain corresponding to the maximum extensibility of entanglement segments, but prior to the unraveling and full extension of chain backbones. In contrast to dilute systems, we find that molten chains become less prone to scission once they unravel from the entanglement network and approach full extension. We suspect this may be due to the effective reduction in intermolecular drag chains experience when their backbones become highly aligned.^20,52,53^

We measure a specific work per scission event w* and decompose it into separate mechanistic contributions associated with conformational alignment, chemical bond dissociation, and scission-induced plasticity. Our analysis shows that activating a chain scission event requires a substantial amount of irreversible dissipation beyond that required to elongate chain backbones into a state of tension. In addition to paying the energy required to break a covalent bond, a chain must also pay the mechanical energy required to plastically redistribute the material near the breaking bond to accommodate the bond separating. This intrinsic plastic work of scission is much larger by multiple orders of magnitude than the intrinsic chemical work required to break covalent bonds for our model melts. Simply put, the efficiency of mechanical degradation in melts appears to be dominated by the nonlinear viscoelastic dissipation of the melt and not by the intrinsic cohesive energy of the backbone bonds. This underscores a need to consider bulk polymer mechanics and rheology when designing efficient mechanical degradation processes.
